# Distribution of variants in multiple vitamin D-related loci (*DHCR7/NADSYN1*, *GC*, *CYP2R1*, *CYP11A1*, *CYP24A1*, *VDR*, *RXRα* and *RXRγ*) vary between European, East-Asian and Sub-Saharan African-ancestry populations

**DOI:** 10.1186/s12263-020-00663-3

**Published:** 2020-03-13

**Authors:** Patrice Jones, Mark Lucock, George Chaplin, Nina G. Jablonski, Martin Veysey, Christopher Scarlett, Emma Beckett

**Affiliations:** 1 School of Environmental & Life Sciences, University of Newcastle, 10 Chittaway Rd, Ourimbah, NSW 2258 Australia; 2 Hunter Medical Research Institute, New Lambton Heights, NSW Australia; 3 Anthropology Department, The Pennsylvania State University, State College, Pennsylvania USA; 4 Hull-York Medical School, University of Hull, HU6 7RX, Hull, UK

**Keywords:** Vitamin D, Polymorphism, UVB, Skin pigmentation

## Abstract

**Background:**

The frequency of vitamin D-associated gene variants appear to reflect changes in long-term ultraviolet B radiation (UVB) environment, indicating interactions exist between the primary determinant of vitamin D status, UVB exposure and genetic disposition. Such interactions could have health implications, where UVB could modulate the impact of vitamin D genetic variants identified as disease risk factors. However, the current understanding of how vitamin D variants differ between populations from disparate UVB environments is limited, with previous work examining a small pool of variants and restricted populations only.

**Methods:**

Genotypic data for 46 variants within multiple vitamin D-related loci (*DHCR7/NADSYN1*, *GC*, *CYP2R1*, *CYP11A1*, *CYP27A1*, *CYP24A1*, *VDR*, *RXRα* and *RXRγ*) was collated from 60 sample sets (2633 subjects) with European, East Asian and Sub-Saharan African origin via the NCBI 1000 Genomes Browser and ALFRED (Allele Frequency Database), with the aim to examine for patterns in the distribution of vitamin D-associated variants across these geographic areas.

**Results:**

The frequency of all examined genetic variants differed between populations of European, East Asian and Sub-Saharan African ancestry. Changes in the distribution of variants in *CYP2R1*, *CYP11A1*, *CYP24A1*, *RXRα* and *RXRγ* genes between these populations are novel findings which have not been previously reported. The distribution of several variants reflected changes in the UVB environment of the population’s ancestry. However, multiple variants displayed population-specific patterns in frequency that appears not to relate to UVB changes.

**Conclusions:**

The reported population differences in vitamin D-related variants provides insight into the extent by which activity of the vitamin D system can differ between cohorts due to genetic variance, with potential consequences for future dietary recommendations and disease outcomes.

## Introduction

Ultraviolet B radiation (UVB; 290–320 nm) exposure is the primary factor influencing vitamin D status in humans, with environmental UVB levels varying considerably by latitude and season. Furthermore, vitamin D status is modulated by variance in vitamin D-associated genes [1, 2], with key genes relating to the production (*DHCR7/NADSYN1)*, binding and transport (*GC*), metabolism (*CYP2R1*, *CYP27A1*, *CYP27B1*, *CYP11A1* and *CYP24A1*), and activation of vitamin D (*VDR* and *RXRα*, *RXRβ*, *RXRγ*) [3]. Both UVB exposure and vitamin D-associated single nucleotide polymorphisms (SNPs) are risk factors for vitamin D insufficiency and many related diseases, such as cardiovascular disease, infectious diseases and cancers [1, 4, 5].

The impact of UVB and vitamin D-related genetics are not merely additive, but may also be interactive. Indeed, there is evidence that the frequency of SNPs in vitamin D-associated genes reflect changes in UVB environment [6–9]. These findings indicate that the functionality of the vitamin D system varies between individuals of differing ethnicities or UVB environments. Genetic differences between populations may also modify vitamin D’s influence on related disease risk [1, 4], warranting further investigation in this area given the current lack of convincing evidence around vitamin D’s roles in many diseases [10]. However, despite an abundance of research into vitamin D-related variants, studies focusing on how the distribution of such variants differs between geographic populations is limited.

The relationship between vitamin D-associated SNPs and skin pigmentation is an important consideration regarding differences between geographically defined populations. Skin pigmentation is an apparent adaptation to differing UVB environments, with darker-pigmented populations originating in areas of high UVB, and lighter-pigmented populations in lower UVB areas [11–13]. However, the genetic architecture underlying skin pigmentation differs even between populations exposed to similar UVB regimes. A key example of this is the fact that parts of Europe and East Asia share similar UVB conditions, but the evolution of lighter skin phenotypes in these populations evolved independently, via different genetic adaptions [14, 15].

Similar geographic patterns may exist in vitamin D-associated SNPs. Both vitamin D and skin pigmentation pathways respond to changes in UVB. Importantly, the vitamin D hypothesis proposes that the reduction of skin pigmentation in early humans migrating out of Africa to areas of lower UVB areas occurred to facilitate vitamin D production [11, 12]. This hypothesis is based on the UVB induced synthesis of vitamin D being dependent on skin pigmentation levels, with competition for UVB absorption existing between pigments and the vitamin D cholesterol precursor. Consequently, lighter-skinned individuals can synthesise up to 30 times more vitamin D than darker-skinned individuals following identical UVB exposure [16].

Our current understanding of how variation in vitamin D-associated genes differs between global populations is limited. Notably, there has been a significant focus on examining vitamin D genetics in Europeans [17–19] with little attention given to other global populations. Therefore, in the present study, a more comprehensive approach has been taken; genotypic data for variants within multiple vitamin D-related genes was collated from 60 sample sets [2633 subjects] with European, East Asian and Sub-Saharan African origin to examine for potential patterns in the geographic distribution of vitamin D-associated SNPs.

## Results

### Validation of European, East Asian and Sub-Saharan African groups with skin pigmentation SNPs

The mean allelic frequencies of *SLC24A5* rs1426654, *SLC45A2* rs16891982 and *OCA2* rs1800414 in derived geographic groups did not deviate from previously reported frequencies in populations of European (EUR), East Asian (EAS) and Sub-Saharan African (AFR) ancestry [20, 21]. rs1426654 and rs16891982 frequency were the highest in EUR (0.99 and 0.91, respectively). Conversely, rs1426654 and rs16891982 were near absent in EAS and AFR (mean frequencies 0.00–0.08; Table 1). Presence of rs1800414 was exclusive to the EAS group (mean frequency 0.59).

**Table 1:** Frequency of skin pigmentation variants in EUR, EAS and AFR groups

		Mean allelic frequency (95% CI)*				
	Sample sets (subjects)	EUR	EAS	AFR	*p*	*r*^2^
*SLC24A5* rs1426654	60 (2598)	0.99 (0.97–1.01)	0.03^a^ (0.00–0.05)	0.08^a^ (0.05–0.10)	6.20e–57	0.99
*SLC45A2* rs16891982	60 (2480)	0.91 (0.89–0.94)	0.01^a^ (- 0.02–0.03)	0.00^a^ (- 0.02–0.03)	4.19e–52	0.98
*OCA2* rs1800414	60 (2633)	0.00^a^ (- 0.03–0.03)	0.59 (0.55–0.62)	0.00^a^ (- 0.03–0.03)	1.09e–36	0.94

*Frequency values notated with the same letter are not significantly different from each other

### Annual UVB levels in European, East Asian and Sub-Saharan African sample set areas

**Fig. 1:**
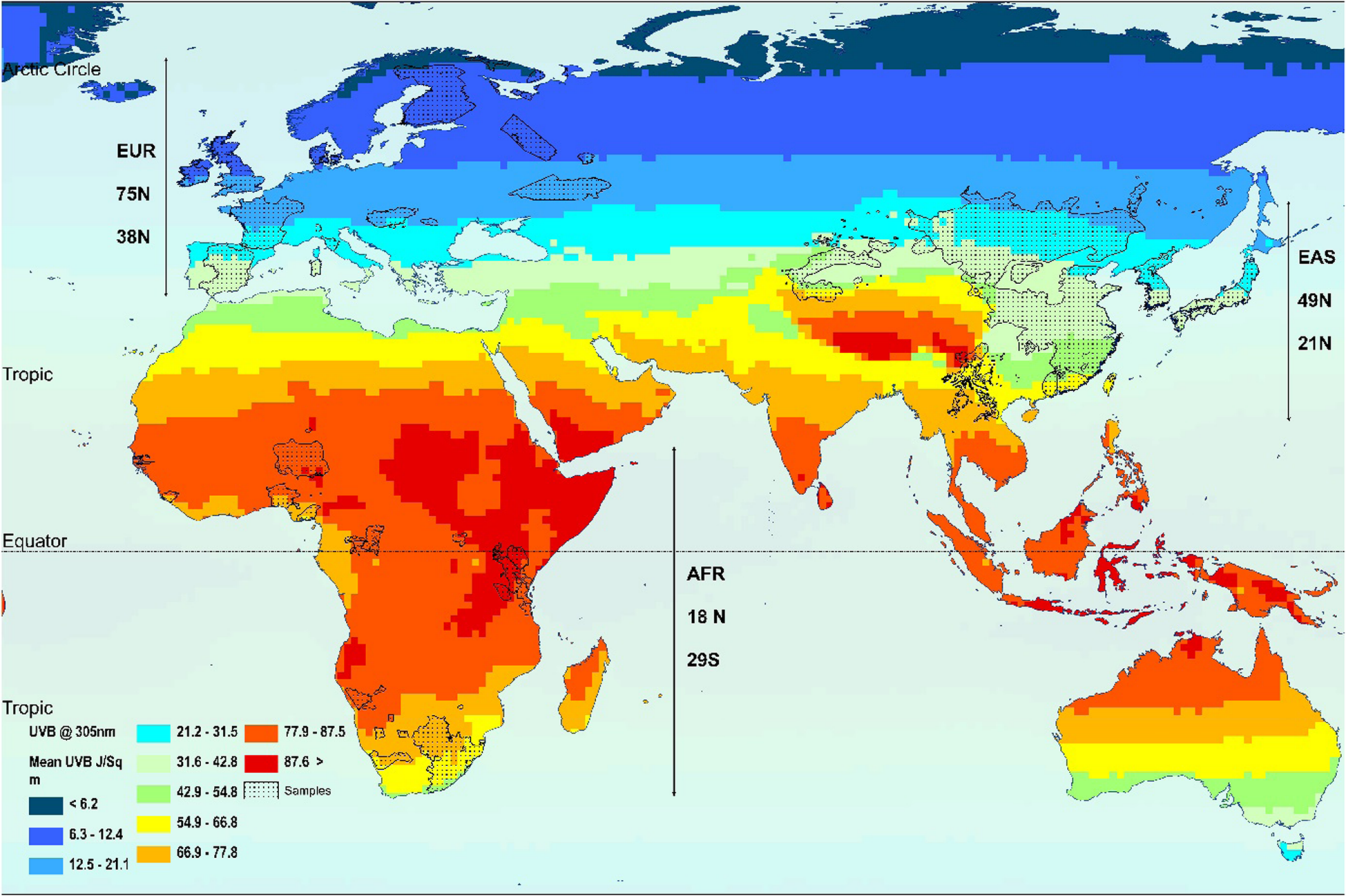
Mean annual UVB levels (surface irradiance at 305 nm) at locations of EUR, EAS and AFR sample sets

Global mean annual UVB levels and sample set locations are shown in Fig. 1, with the highest mean annual UVB levels found in AFR locations followed by EAS and EUR sample set locations as expected (82.2 vs. 48.1 vs. 18.4 Mw/m^2^/nm respectively). Intergroup comparisons found significant differences between all geographic areas for annual UVB levels (*p* < 0.001).

### Distribution of vitamin D production/transport-related variants (NADSYN1/DHCR7 and GC) across European, East Asian and Sub-Saharan African groups

Sixteen variants in genes involved in vitamin D production (*NADSYN1/DHCR7*) and transport (*GC*) were examined, eight within the *NADSYN1/DHCR7* loci and eight within *GC* (Table 2).

**Table 2:** Frequency of *NADSYN1/DHCR7* and *GC* variants in EUR, EAS and AFR groups

		Mean allelic frequency (95% CI)*					
	Sample sets (subjects)	EUR	EAS	AFR	*p*	*r*^2^	Distribution pattern^#^
*NADSYN1/DHCR7*							
rs3750997	60 (2356)	0.30 (0.25–0.36)	0.58^a^ (0.52–0.63)	0.65^a^ (0.59–0.70)	3.18e–12	0.59	EUR≠AFR and EAS
rs1790325	45 (1854)	0.95 (0.89–1.02)	0.73^a^ (0.66–0.79)	0.57^a^ (0.50–0.63)	9.90e–10	0.61	EUR ≠ AFR and EAS
rs11603330	60 (2356)	0.69 (0.64–0.73)	0.35 (0.31–0.40)	0.11 (0.07–0.16)	7.09e–26	0.86	EUR ≠ AFR ≠ EAS
rs7928249	34 (2293)	0.29 (0.22–0.36)	0.66^a^ (0.58–0.74)	0.63^a^ (0.56–0.70)	1.06e-08	0.67	EUR ≠ AFR and EAS
rs12800438	60 (2352)	0.30 (0.24–0.35)	0.63^a^ (0.57–0.68)	0.66^a^ (0.61–0.71)	3.04e–14	0.65	EUR ≠ AFR and EAS
rs7944926	60 (2356)	0.30 (0.25–0.34)	0.63 (0.58–0.67)	0.86 (0.82–0.91)	1.68e–24	0.85	EUR ≠ AFR ≠ EAS
rs3794060	60 (2356)	0.70 (0.66–0.75)	0.37 (0.33–0.42)	0.11 (0.07–0.15)	5.87e–26	0.87	EUR ≠ AFR ≠ EAS
rs12280295	60 (2356)	0.00^a^ (− 0.04–0.04)	0.00^a^ (− 0.04–0.04)	0.23 (0.19–0.26)	1.57e–13	0.63	AFR ≠ EUR and EAS
*GC*							
rs7041	60 (2354)	0.58 (0.55–0.61)	0.28 (0.25–0.31)	0.10 (0.07–0.13)	2.17e–30	0.91	EUR ≠ AFR ≠ EAS
rs4364228	60 (2354)	0.09^a^ (0.06–0.13)	0.12^a^ (0.09–0.15)	0.45 (0.42–0.48)	2.16e–24	0.85	AFR ≠ EUR and EAS
rs222047	60 (2356)	0.58 (0.55–0.61)	0.23 (0.20–0.26)	0.12 (0.09–0.14)	4.09e–31	0.91	EUR ≠ AFR ≠ EAS
rs3737549	60 (2356)	0.00 (− 0.03–0.03)	0.14^a^ (0.11–0.17)	0.22^a^ (0.19–0.25)	6.72e–14	0.64	EUR ≠ AFR and EAS
rs222016	60 (2327)	0.85 (0.82–0.88)	0.62 (0.59–0.65)	0.37 (0.34–0.40)	2.20e–30	0.91	EUR ≠ AFR ≠ EAS
rs222020	60 2356	0.85 (0.82–0.88)	0.62 (0.59–0.65)	0.36 (0.33–0.39)	1.54e–30	0.91	EUR ≠ AFR ≠ EAS
rs843006	60 (2355)	0.83 (0.80–0.86)	0.62 (0.59–0.65)	0.31 (0.28–0.35)	6.98e–30	0.90	EUR ≠ AFR ≠ EAS
rs705117	60 (2356)	0.84 (0.81–0.88)	0.50 (0.46–0.53)	0.17 (0.14–0.20)	6.42e–36	0.94	EUR ≠ AFR ≠ EAS

*Frequency values notated with the same letter are not significantly different from each other

^#^EUR ≠ AFR ≠ EAS; allelic frequency differed between all geographic groups, EUR ≠ AFR and EAS; allelic frequency differed between EUR and both AFR and EAS, AFR ≠ EUR and EAS; allelic frequency differed between AFR and both EUR and EAS

The frequencies of all *NADSYN1/DHCR7* variants varied by geographic group (*p* < 0.0001, *r*^2^ 0.59–0.87). Patterns of distribution varied by SNP (Table 2). For *NADSYN1/DHCR7* variants rs11603330, rs7944926 and rs3794060, allelic frequency differed between all geographic groups, with their distribution coinciding with changes in environmental UVB. rs7944926 increased in areas of increased environmental UVB (i.e. frequency highest in AFR, lowest in EUR), whilst rs11603330 and rs3794060 decreased with increased UVB levels (i.e. frequency lowest in AFR, highest in EUR).

Four other *NADSYN1/DHCR7* variants, rs3750997, rs1790325, rs7928249 and rs12800438, frequencies differed in EUR compared to EAS and AFR. rs3750997, rs7928249 and rs12800438 frequencies were increased in EAS and AFR, compared to EUR, with the inverse relationship observed for rs1790325. Another *NADSYN1/DHCR7* variant, rs12280295, was near absent in the EUR and EAS (mean frequencies of 0.00), with higher frequency in AFR (0.23). Considering these distribution patterns together, there was no apparent trend for *NADSYN1/DHCR7* polymorphisms to be in higher in one geographic region over another.

The allelic frequency of all examined *GC* genotypes varied by geographic group (*p* < 0.0001, *r*^2^ 0.64–0.94). The largest effect was observed for rs705117 (*p* < 0.0001, *r*^2^ 0.94), with the frequency of this variant differing between all geographic regions, and decreasing in geographic areas of increasing UVB (EUR 0.84, EAS 0.50 and AFR 0.17). Interestingly, five other *GC* variants followed this distribution pattern (rs7041, rs222047, rs222016, rs222020, rs843006 and rs705117). Another *GC* variant, rs4364228 had reduced frequencies in EUR (0.09) and EAS (0.12) compared to AFR (0.45), and a further variant, rs3737549, was shown to absent in the EUR group (0.00), but increasingly present in EAS and AFR (0.14 and 0.22, respectively; Table 2). Considered together, frequencies of examined *GC* variants were the highest in either EUR or AFR groups, with high frequencies in EAS uncommon.

### Distribution of variants in vitamin D metabolism genes (CYP11A1, CYP24A1, CYP27A1 and CYP2R1) across European, East Asian and Sub-Saharan African groups

Fourteen cytochrome P450 (CYP) variants fit the inclusion criteria (two in *CYP11A1*, five each in *CYP24A1* and *CYP27A1* and two in *CYP2R1*). Allelic frequency of all 14 variants varied by geographic groups (*p* < 0.0001; Table 3).

**Table 3:** Frequency of *CYP11A1*, *CYP24A1*, *CYP27A1* and *CYP2R1* variants in EUR, EAS and AFR groups

	Sample sets (subjects)	Mean allelic frequency (95% CI)*					Distribution pattern^#^
		EUR	EAS	AFR	*p*	*r*^2^	
*CYP11A1*							
rs11632698	60 (2357)	0.57 (0.54–0.60)	0.20^a^ (0.17–0.23)	0.20^a^ (0.17–0.23)	3.37e–25	0.86	EUR ≠ AFR and EAS
rs2073475	60 (2352)	0.16 (0.13–0.16)	0.45 (0.42–0.48)	0.58 (0.55–0.61)	1.89e–27	0.88	EUR ≠ AFR ≠ EAS
*CYP24A1*							
rs3787557	60 (2349)	0.13 (0.11–0.16)	0.27 (0.24–0.29)	0.00 (− 0.02–0.03)	3.55e–22	0.82	EUR ≠ AFR ≠ EAS
rs927650	60 (2380)	0.47 (0.44–0.49)	0.27 (0.25–0.30)	0.18 (0.16–0.21)	4.52e–22	0.82	EUR ≠ AFR ≠ EAS
rs912505	60 (2356)	0.21 (0.18–0.25)	0.62 (0.59–0.65)	0.45 (0.41–0.48)	1.04e–23	0.84	EUR ≠ AFR ≠ EAS
rs2762929	69 (2356)	0.58 (0.55–0.61)	0.30^a^ (0.26–0.33)	0.25^a^ (0.22–0.29)	1.40e–20	0.79	EUR ≠ AFR and EAS
rs4809956	45 (1854)	0.81 (0.77–0.86)	0.43^a^ (0.39–0.47)	0.40^a^ (0.36–0.44)	2.23e–18	0.85	EUR ≠ AFR and EAS
*CYP27A1*							
rs7568196	60 (2355)	0.40 (0.35-0.44)	0.06^a^ (0.02–0.11)	0.18^a^ (0.13–0.22)	3.20e–14	0.65	EUR ≠ AFR and EAS
rs4674338	60 (2301)	0.58 (0.56–0.61)	0.93 (0.91–0.96)	0.74 (0.71–0.76)	1.94e–27	0.88	EUR ≠ AFR ≠ EAS
rs13013510	60 (2356)	0.51 (0.47–0.54)	0.19 (0.15–0.22)	0.65 (0.62–0.69)	5.91e–26	0.87	EUR ≠ AFR ≠ EAS
rs691414	60 (2356)	1.00^a^ (0.98–1.02)	1.00^a^ (0.98–1.02)	0.78 (0.76–0.79)	1.31e–28	0.89	AFR ≠ EUR and EAS
rs692290	60 (2356)	1.00^a^ (0.98–1.02)	1.00^a^ (0.98–1.02)	0.60 (0.58–0.62)	2.20e–42	0.96	AFR ≠ EUR and EAS
*CYP2R1*							
rs16930625	60 (2356)	0.08^a^ (0.06–0.11)	0.12^a,b^ (0.10–0.15)	0.19^b^ (0.17–0.21)	1.03e–07	0.41	AFR ≠ EUR
rs11023374	60 (2342)	0.28 (0.26–0.31)	0.09^a^ (0.06–0.11)	0.02^a^ (− 0.01–0.04)	1.35e–20	0.79	EUR ≠ AFR and EAS

*Frequency values notated with the same letter are not significantly different from each other

^#^EUR ≠ AFR ≠ EAS; allelic frequency differed between all geographic groups, EUR ≠ AFR and EAS; allelic frequency differed between EUR and both AFR and EAS, AFR ≠ EUR and EAS; allelic frequency differed between AFR and both EUR and EAS, allelic frequency differed between AFR and EUR.

Two *CYP11A1* variants varied in frequency by geographic group (rs11632698 and rs2073475; *p* < 0.0001, *r*^2^ 0.86 and 0.88, respectively) but displayed different distribution patterns across geographic groups. The distribution of *CYP11A1* rs2073475 coincided with increasing UVB (EUR 0.16, EAS 0.45 and 0.58). *CYP11A1* rs11632698 frequency significantly differed in EUR compared to EAS and AFR (mean frequency of 0.57 in EUR and 0.20 in EAS and AFR).

Five *CYP24A1* variant frequencies varied by geographic group (rs3787557, rs927650, rs912505, rs2762929 and rs4809956, *p* < 0.0001, *r*^2^ 0.82–0.85). For three variants, frequencies differed between all geographic groups (rs3787557, rs927650 and rs912505). A potential UVB-dependent trend in rs927650 was noted (frequencies of 0.47, 0.27 and 0.18 in EUR, EAS and AFR groups respectively). For another two variants, rs2762929 and rs4809956, frequency was significantly higher in EUR (rs2762929 0.58, rs4809956 0.81) compared to EAS and AFR groups (rs2762929 0.22–0.33, rs4809956 0.36–0.47). Examining these distribution patterns together, frequencies of *CYP24A1* and *CYP27A1* variants tended to be the highest in EUR or EAS groups.

Two of the 5 examined *CYP27A1* variants, rs691414 and rs692290, appeared to be fixed in EUR and EAS (mean allelic frequencies of 1.00). Conversely, frequencies were significantly reduced in AFR (rs691414; 0.78 and rs692290; 0.60). These variants had the largest effect sizes of examined *CYP27A1* variants (*p* < 0.0001, rs691414 *r*^2^ 0.89, rs692290; *r*^2^ 0.96). The remaining examined *CYP27A1* variants displayed differing patterns in allelic frequency. rs7568196 had low frequencies in EAS and AFR (0.02–0.22), with increased frequency in EUR (0.40). Frequency of rs13013510 and rs4674338 were significantly different in all geographic groups, with the highest frequency for rs13013510 reported in AFR (0.65), and EAS for rs4674338 (0.93). Interestingly, despite differing distribution patterns observed for *CYP27A1* variants, there was a trend for frequencies of these variants to be the highest in EUR and EAS over AFR.

The frequencies of *CYP2R1* variants (rs16930625 and rs11023374) differed by geographic group (*p* < 0.0001, rs16930625; r^2^ 0.41 rs11023374; r^2^ 0.79), although there was no trend for *CYP2R1* variants to be higher in one geographic region over others. rs16930625 had low frequencies in all groups (0.06–0.21), but was higher in AFR compared to EUR. rs11023374 had a lower frequency in EAS and AFR (0.01–0.11), compared to EUR (0.28).

### Distribution of variants in genes relating to vitamin D activity (VDR, RXRα and RXRγ) across European, East Asian and Sub-Saharan African groups

Sixteen variants in vitamin D-related nuclear receptor genes were examined (five *VDR*, seven *RXRα* and four *RXRγ*; Table 4).

**Table 4:** Frequency of *VDR*, *RXRα* and *RXRγ* variants in EUR, EAS and AFR groups

	Sample sets (subjects)	Mean allelic frequency (95% CI)*					Distribution pattern^#^
		EUR	EAS	AFR	*p*	*r*^2^	
*VDR*							
rs886441	60 (2356)	0.18 (0.16–0.21)	0.05 (0.02–0.07)	0.41 (0.38–0.43)	1.66e–27	0.88	EUR ≠ AFR ≠ EAS
rs2283342	60 (2356)	0.26 (0.22–0.29)	0.49 (0.43–0.55)	0.00 (− 0.04–0.05)	6.70e–19	0.76	EUR ≠ AFR ≠ EAS
rs2107301	60 (2356)	0.32 (0.29–0.35)	0.67 (0.63–0.70)	0.13 (0.10–0.16)	1.91e–30	0.91	EUR ≠ AFR ≠ EAS
rs4334089	60 (2356)	0.73 (0.68–0.77)	0.57 (0.52–0.61)	0.35 (0.30–0.39)	1.95e–16	0.71	EUR ≠ AFR ≠ EAS
rs4516035	60 (2355)	0.43 (0.41–0.45)	0.03^a^ (0.00–0.05)	0.03^a^ (0.01–0.05)	3.68e–34	0.93	EUR ≠ AFR and EAS
*RXRα*							
rs1805343	45 (1853)	0.66^a^ (0.63–0.69)	0.65^a^ (0.63–0.68)	0.17 (0.14–0.19)	6.81e–29	0.95	AFR ≠ EUR and EAS
rs1805352	44 (1822)	0.69^a^ (0.66–0.72)	0.75^a^ (0.72–0.78)	0.26 (0.23–0.29)	2.82e–27	0.95	AFR ≠ EUR and EAS
rs10881582	46 (1881)	0.74^a^ (0.71–0.77)	0.81^a^ (0.78–0.83)	0.25 (0.22–0.28)	1.04e–30	0.96	AFR ≠ EUR and EAS
rs3118571	45 (1854)	0.65^a^ (0.62–0.68)	0.69^a^ (0.66–0.72)	0.12 (0.09–0.15)	9.27e–30	0.96	AFR ≠ EUR and EAS
rs3818740	45 (1853)	0.40^a,b,c^ (0.29–0.52)	0.43^a,b,c^ (0.32–0.53)	0.59^a,b,c^ (0.48–0.70)	3.85e–02	0.10	-
rs731516	45 (1852)	1.00^a^ (0.98–1.02)	1.00^a^ (0.98–1.02)	0.59 (0.57–0.61)	1.20e–31	0.96	AFR ≠ EUR and EAS
rs7040434	45 (1854)	0.00^a^ (− 0.01–0.02)	0.00^a^ (− 0.01–0.01)	0.53 (0.52–0.54)	4.26e–44	0.99	AFR ≠ EUR and EAS
*RXRγ*							
rs283695	60 (2356)	0.44 (0.40–0.47)	0.77^a^ (0.73–0.80)	0.85^a^ (0.82–0.89)	8.45e–25	0.85	EUR ≠ AFR and EAS
rs12069160	60 (2356)	0.94^a^ (0.92–0.96)	0.65 (0.62–0.67)	0.92^a^ (0.90–0.95)	2.69e–24	0.85	EAS ≠ AFR and EUR
rs10800098	60 (2345)	0.05^a^ (0.02–0.08)	0.33 (0.30–0.36)	0.03^a^ (0.00–0.06)	8.40e–21	0.80	EAS ≠ AFR and EUR
rs10489745	60 (2343)	0.09^a^ (0.07–0.12)	0.42 (0.40–0.45)	0.03^a^ (0.01–0.05)	3.57e–33	0.92	EAS ≠ AFR and EUR

*Frequency values notated with the same letter are not significantly different from each other

^#^EUR ≠ AFR ≠ EAS; allelic frequency differed between all geographic groups, EUR ≠ AFR and EAS; allelic frequency differed between EUR and both AFR and EAS, AFR ≠ EUR and EAS; allelic frequency differed between AFR and both EUR and EAS, EAS ≠ AFR and EUR; allelic frequency differed between EAS and both EUR and AFR

The allelic frequencies of all examined *VDR* variants varied by geographic group (rs886441, rs2283342, rs2107301, rs4334089 and rs4516035; *p* < 0.0001, *r*^2^ 0.71–0.93). The greatest effect size was for rs4516035 (*p* < 0.0001, *r*^2^ 0.93), which had reduced frequencies in AFR and EAS (0.03), compared to EUR (0.43). *VDR* rs886441, rs2283342, rs2107301 and rs4334089 allelic frequencies differed between all geographic groups. Only rs4334089 appeared to have a UVB relationship, with frequency decreasing in areas of increasing UVB.

Six of the seven examined *RXRα* variants varied by the examined geographic groups (rs1805343, rs1805352, rs10881582, rs3118571, rs731516 and rs7040434; *p* < 0.0001; *r*^2^ 0.95–0.99). Interestingly, these six *RXRα* variants followed the same distribution pattern, with differences in AFR when compared to EUR and EAS. For five variants (rs1805343, rs1805352, rs10881582, rs3118571 and rs731516), the allelic frequency was reduced in AFR compared to EAS and EUR. Notably, *RXRα* rs731516 was fixed in EUR and EAS (mean frequency of 1.0), with reduced frequency in AFR (0.59). rs7040434 was absent in EUR and EAS (0.00) but not AFR (0.53; *r*^2^ 0.99).

Four *RXRγ* variants varied by geographic group (rs283695, rs12069160, rs10800098 and rs10489745; *p* < 0.0001, *r*^2^ 0.80–0.92). Frequencies of three variants (rs12069160, rs10800098 and rs10489745) did not differ between EUR and AFR, but frequency differed in EAS. The rs283695 variant had increased frequencies in EUR and AFR (0.77 and 0.85 respectively) compared to EUR (0.44).

There was no trend for examined *VDR* and *RXRγ* variants to be higher in specific geographic groups, although frequencies of examined *RXRα* variants appeared to be the highest in either EUR or EAS. However, genotypic data for the *RXRα* variants were only available for 44–46 of the 60 included sample sets, and unavailable data were mostly from EUR and AFR sample sets, so this may have influenced results.

## Discussion

This study demonstrates that variant frequency in multiple vitamin D-associated genes (*VDR*, *RXRα*, *RXRγ*, *GC*, *CYP2R1*, *CYP27B1*, *CYP24A1*, *CYP11A1* and *DHCR7/NADSYN1)* varies by environmental UVB and ancestry. For many SNPs, frequency followed a trend to either decrease or increase in geographic regions of increasing environmental UVB. However, several SNPs displayed a population-specific pattern that cannot be explained by changes in UVB levels alone. This provides insights into the extent to which vitamin D regulation differs by cohort, and may have consequences for public health recommendations and disease outcomes.

The reported geographic patterns in the frequency of SNPs in *CYP* genes and *RXRα* are novel findings. Whilst such variants have been examined previously in differing cohorts, details into how the distribution of these variants differs by ancestry has not been highlighted. CYP2R1 and CYP27A1 enzymatically activate vitamin D, and formation of the excretory form is enzymatically regulated by CYP24A1. CYP11A1 is highly expressed in the skin and represents an important alternative vitamin D metabolism pathway [3, 22]. As such, genetic variance in these pathways may influence vitamin D status and homeostasis.

Multiple *RXRα* variants displayed similar frequencies in EUR and EAS populations, potentially related to a broad reduction in UVB in Europe and East Asia compared to Sub-Saharan Africa. RXR are the most common subunit forming heterodimers with VDR, but little is known about the influence of *RXR* variants on vitamin D activity [23]. Expression of the RXRα subtype is particularly high in skin, and therefore SNPs could be of functional relevance to UVB-induced vitamin D activity [24, 25]. However, other UVB-related roles of retinoids and vitamin A derivatives in the skin should be considered, including involvement in circadian rhythm and photo-protection [26].

*DHCR7/NADSYN1*, *VDR, RXRγ*, *CYP2R1*, *CYP24A1* and *CYP11A1* variants did not display clear patterns of geographic distribution, likely reflecting diverse functional consequences. However, the majority of examined variants reside within introns or untranslated regions. Therefore, linkage disequilibrium of these variants with nearby functional variants needs to be considered.

It was hypothesised that selection of vitamin D-related SNPs would parallel geographic selection for skin pigmentation. The reported associations support this and indicate vitamin D SNPs display population-specific patterns, with genetic differences observed between populations which did not reflect increases and/or decreases in ancestral UVB environments. These population-specific patterns could coincide with migration patterns, as in the case of variants underlying skin pigmentation [14, 15] and support a link between vitamin D and the evolution of lighter skin, with further examination into this association warranted. Notably, evidence of positive selection for *DHCR7/NADSYN1* variants has been reported; however, evidence of selection was not found for other examined vitamin D-related genes (*CYP2R1* and *GC*), possibly due to selection taking place at an earlier time than examined, and/or in other vitamin D-associated genes, such as *CYP27B1*, *CYP24A1* or *VDR*.

Many of the reported associations support previously reported frequency patterns in *GC*, *VDR* and *DHCR7/NADSYN1* variants [6, 7, 27, 28]. *GC* rs7041 is a genetic determinant of vitamin D status, with a negative association between frequency and latitude reported [28, 29]. Here, similar latitudinal/UVB clines for several additional *GC* variants were observed. Of these, rs705117 and rs222020 have been linked to vitamin D status [30, 31]. Latitudinal clines in *VDR* SNPs have been observed, although these associations were limited to the Africa-Europe axis [6–8]. Potential latitudinal clines exist for several *VDR* variants examined here along this axis, but not when considering the East Asian populations. Several examined *DHCR7/NADSYN1* variants (rs12800438, rs7944926, rs3794060, rs12280295) are part of a large haplotype block previously noted to have high frequency in Europeans and North East Asians [27]. Here multiple additional variants in this locus that differed in frequency between populations that may be functionally relevant were identified.

Strengths of this study include the collation of numerous cohorts from three genetically distinct populations exposed to differing UVB regimes and the simultaneous examination of multiple vitamin D-associated variants. However, the analysis was limited by data availability. Furthermore, the inclusion of multiple cohorts from the same area (e.g. multiple Italian and Han cohorts) might have resulted in over-representation of sub-populations in derived geographic groups.

This data is interesting from a human evolution perspective but also has relevance for public health recommendations and understanding disease risk. Vitamin D insufficiency is more likely in darker-skinned individuals, attributed to diminished synthesis of the vitamin due to pigmentation [5, 32, 33]. However, variants displaying apparent interethnic differences in frequency may also contribute to population differences in vitamin D status, and therefore current global and national dietary recommendations for this vitamin may not meet the needs of all populations equally. Further, numerous SNPs in vitamin D pathways have been identified as risk factors for multiple adverse health conditions [1, 4]. Given that variant frequency appears to vary by ancestry, disease risk factors could be population specific. A further possibility is that risks conferred by vitamin D SNPs may change depending on environmental factors, such as UVB exposure, with these concepts requiring further examination.

## Conclusions

This study reports population differences for gene variants within multiple vitamin D-related loci that have not been explored previously. A key finding was that the frequency of many of these vitamin D variants are population-specific, and do not reflect changes in ancestral UVB environments. These population differences provide insight into the extent to which vitamin D metabolism and activity may vary between populations of different ancestry via genetic variance in numerous vitamin D-related genes. Given multiple SNPs within examined loci have been identified as disease risk factors, further examination of identified gene variants displaying interethnic differences in frequency and their potential relevance to disease outcomes is warranted.

## Methods

NCBI 1000 Genomes Browser [34] and ALFRED (Allele Frequency Database) [35] databases were searched for variants in vitamin D-related genes; *VDR*; encoding for the vitamin D receptor; *RXRα*, *RXRβ*, and *RXRγ*; retinoid X receptor subtypes, *GC*; vitamin D binding protein, *CYP2R1*, *CYP24A1*, *CYP11A1*, *CYP27A1 and CYP27B1*; vitamin D hydroxylases, and *DHCR7/NADSYN1*; 7-dehydrocholesterol reductase/NAD(+) synthetase (examined together due their close positioning on the genome). Genotypic data was available for 170 variants in these genes. Variants were ranked by population differentiation, using fixation indices (FST) provided by ALFRED. Variants with the highest FST (top 30%; FST ≥ 0.13) were included in analyses, resulting in the inclusion of 51 variants in eight loci. A further four variants with unknown functional consequences were excluded (as per dbSNP - www.ncbi.nlm.nih.gov/snp), resulting in the analysis of 46 variants in *VDR*, *RXRα*, *RXRβ*, *GC*, *CYP2R1*, *CYP24A1*, *CYP11A1* and *DHCR7/NADSYN1* (Table 5). No *RXRβ* or *CYP27B1* variants fit the inclusion criteria.

**Table 5:** Vitamin D-associated variants included in the study

Gene/loci	ID number	Fixation index	Minor allele frequency	Variant allele	Function class^#^
*CYP11A1*	rs11632698	0.18	0.35	G	Intron
	rs2073475	0.13	0.38	A	nearGene-5
*CYP24A1*	rs3787557	0.13	0.12	C	Intron
	rs927650	0.14	0.34	T	Intron
	rs912505	0.14	0.39	G	Intron
	rs2762929	0.15	0.39	T	Intron
	rs4809956	0.18	0.40	C*	Intron
*CYP27A1*	rs7568196	0.14	0.25	A	Intron
	rs4674338	0.17	0.25	G	Intron
	rs13013510	0.18	0.43	G	Intron
	rs691414	0.30	0.06	C	Intron
	rs692290	0.38	0.11	G	Intron
*CYP2R1*	rs16930625	0.14	0.15	G	5′ Prime UTR
	rs11023374	0.17	0.17	C	Intron
*DHCR7/NADSYN1*	rs3750997	0.16	0.42	T*	Intron
	rs1790325	0.18	0.26	A	Intron
	rs11603330	0.18	0.35	A	Intron
	rs7928249	0.20	0.42	A*	nearGene-5
	rs12800438	0.16	0.40	G*	Intron
	rs7944926	0.22	0.35	A	Intron
	rs3794060	0.23	0.35	T	Intron
	rs12280295	0.53	0.05	C	Intron
*GC*	rs7041	0.14	0.38	G	Missense
	rs4364228	0.14	0.17	G	Intron
	rs222047	0.16	0.38	G	Intron
	rs3737549	0.16	0.10	T	Intron
	rs222016	0.17	0.34	A*	Intron
	rs222020	0.17	0.34	T*	Intron
	rs843006	0.19	0.36	G	Intron
	rs705117	0.25	0.42	A	Intron
*RXRα*	rs1805343	0.16	0.48	A	Intron
	rs1805352	0.16	0.47	A*	Intron
	rs10881582	0.18	0.38	G*	Intron
	rs3118571	0.19	0.48	A	Intron
	rs3818740	0.20	0.38	T*	Intron
	rs731516	0.43	0.11	A	Intron
	rs7040434	0.51	0.15	C	Intron
*RXRγ*	rs283695	0.14	0.34	A	Intron
	rs12069160	0.15	0.14	T	Intron
	rs10800098	0.16	0.12	A	Intron
	rs10489745	0.17	0.17	C	Intron
*VDR*	rs886441	0.13	0.23	C	Intron
	rs2283342	0.13	0.19	C	Intron
	rs2107301	0.16	0.34	T	Intron
	rs4334089	0.17	0.41	G*	Intron
	rs4516035	0.18	0.18	C	2KB upstream

*Indicates the variant allele was also the major allele

^#^Functional class recorded and defined by dpSNP—see www.ncbi.nlm.nih.gov/variation/docs/glossary/

Allelic frequencies of included variants were grouped based on European (EUR), East Asian (EAS) or Sub-Saharan African (AFR) ancestry and current residence (Table 6). The United Nations (UN) Geoscheme [37] was used to define the populations included in EUR, EAS and AFR groups. An adjustment was made to include only European Russia in Eastern Europe; defined as the western part of the Russian Federation bordered by the Ural Mountains range [38].

**Table 6:** Sample sets in derived EUR, EAS and AFR geographic groups

Europeans (EUR)				
ALFRED				
Sample set	*n*	Latitude^^^	Sample ID^#^	Data source*
Danes	51	55.0–58.0° N	SA000007H	[35]
Finns	36	60.0–75.0° N	SA000018J	[35]
Orcadian	16	59.0° N	SA001508O	[36]
Irish	116	51.0–56.0° N	SA000057M	[35]
French	28	46.0° N	SA001503J	[36]
Basque (France)	24	43.0° N	SA001504K	[36]
Hungarians	92	45.5–48.5° N	SA002023H	[35])
Italian (Tuscan)	8	43.0° N	SA001507N	[36]
Italian (Bergamo)	14	46.0° N	SA002255O	[36]
Sardinian	28	40.0° N	SA001505L	[36]
Russians (Vologda)	25	61.0° N	SA001510H	[36]
Russians (Archangel’sk)	34	63.0–64.5° N	SA001530J	[35]
Chuvash	42	54.5–56.5° N	SA000491O	[35]
Adygei (Krasnodar)	54	44.0–45° N	SA000017I	[8]
1000 Genomes				
British from England and Scotland (GBR)	91	49.8–59.5° N		
Finnish in Finland (FIN)	99	60.0–75.0° N		
Iberians in Spain (IBS)	107	36.0–43.5° N		
Toscani in Italia (TSI)	107	38.0–47.0° N		
Total	972			
East Asians (EAS)				
ALFRED				
Sample set	*n*	Latitude	Sample ID^#^	Data source*
Ami (Taiwan)	40	22.5–24.0° N	SA000002C	[35]
Atayal (Taiwan)	41	21.8–25.5° N	SA000021D	[35]
Dai (China)	10	21.0° N	SA001493R	[36]
Daur (China)	10	48.0–49.0° N	SA001488V	[36]
Han (China)	45	36.0–39.0° N	SA001483Q	[36]
Hezhen (China)	10	47.0–48.0° N	SA001490O	[36]
Japanese	29	38.0° N	SA002260K	[36]
Koreans	53	34.5–43.0° N	SA003027M	[35]
Lahu (China)	10	22.0° N	SA001494S	[36]
Miao (China)	10	28.0° N	SA001486T	[36]
Naxi (China)	10	26.0° N	SA001496U	[36]
Oroqen (China)	10	48.0–53.0° N	SA001487U	[36]
She (China)	10	27.0° N	SA001495T	[36]
Tu (China)	10	36.0° N	SA001497V	[36]
Tujia (China)	10	29.0° N	SA001484R	[36]
Uygur (China)	10	44.0° N	SA001492Q	[36]
Xibo (China)	9	43.0–44.0° N	SA001491P	[36]
Yizu (China)	10	28.0° N	SA001485S	[36]
Hakka (Taiwan)	43	22.0–35.0° N	SA000003D	[35]
Mongolian (China)	10		SA001489W	[36]
1000 Genomes				
Chinese Dai in Xishuangbanna, China (CDX)	93	21.0–28.0° N		
Han Chinese in Beijing, China (CHB)	103	22.0–40.0° N		
Han Chinese South, China (CHS)	105	22.0–40.0° N		
Japanese in Tokyo, Japan (JPT)	104	30.0–46.0° N		
Total	795			
Sub-Saharan Africans (AFR)				
ALFRED				
Sample set	*n*	Latitude	Sample ID^#^	Data source*
Bantu (SA)	8	22.0–29.0° S	SA001818S	[36]
Bantu (Kenya)	12	3.0° S	SA001819T	[36]
San (Nambia)	7	21.0° S	SA001469U	[36]
Biaka (C. African Republic)	35	4.0° N	SA001465Q	[36]
Hausa (Nigeria)	39	7.0–18.0° N	SA000100B	[35]
Ibo (Nigeria)	48	5.0–7.0° N	SA000099S	[35]
Mbuti (Demographic Republic of the Congo)	19	1.0° N	SA004361O	[35]
Yoruba (Nigeria)	25	6.0–10.0° N	SA001468T	[36]
Chagga (Tanzania)	45	2.5–3.5° S	SA000487T	[35]
Masai (E Africa)	22	1.0° N–6.0° S	SA000854R	[35]
Sandawe (Tanzania)	39	4.0–7.0° S	SA004366T	[35]
Zaramo (Tanzania)	39	4.0–11.0° S	SA004367U	[35]
Mandenka (Senegal)	24	12.0° N	SA001467S	[36]
1000 Genomes				
Esan in Nigeria (ESN)	99	4.0–12.0° N		
Gambian in Western Division, Mandinka (GWD)	113	7.0–23.0° N		
Yoruba in Ibadan, Nigeria (YRI)	108	6.0–10.0° N		
Luhya in Webuye, Kenya (LWK)	99	1.0° N–3.0° S		
Mende In Sierra Leone (MSL)	85	4.0–10° N		
Total	866			

^#^Sample IDs relate to only sample sets collated from ALFRED database

*Source of sample sets collated from ALFRED; either original sample sets (i.e. only published on ALFRED by the host (Kidd KK.)) [8], or HGDP-CEPH samples [10]

Multiple sample sets from a defined population (e.g. Han Chinese) were included provided there was sufficient evidence they were not duplicates. In the case of duplicates, the most recent data was used. Only sample sets with available genotypic data for all variants used to validate ancestry (described below) were included. Supplementary material 1 outlines available data for each vitamin D-related variant.

Latitude and longitude were recorded for all sample sets. EUR, EAS and AFR groups represented differing latitudinal ranges, with a range of 75° N–38° N for EUR, 49° N–21° N for EAS, 18° N–29° S for AFR (Table 6). These coordinates were used to collect information on the UVB levels in the three geographical regions following previously published methods [39]. Daily noontime surface irradiance data for 305 nm (Mw/m^2^/nm) was collected from the NASA Nimbus-7 Total Ozone Mapping Spectrometer for the total available period (15 years; 1978–1993), accessed via NASA’s web application, Giovanni [40]. Data was collected for UVB cells covering a half-degree latitude by longitude area within sample set locations and then used to calculate mean UVB levels for sample set location and then for each geographic region. The 305 nm wavelength was chosen as this was the shortest and most relevant of published available wavelengths to vitamin D UVB-synthesis [2]. Further information on UVB data collected for sample sets can be found in the supplementary material 1.

Allelic frequencies of three skin pigmentation-related variants, *SLC24A5* rs1426654, *SLC45A2* rs16891982 and *OCA2* rs1800414, were used to assess if derived geographic groups accurately represented geographic regions with distinct ancestral skin pigmentation [20, 21]. The mean allelic frequencies for derived geographic groups were compared against previously reported frequencies for European, East Asian and Sub-Saharan originating populations. rs1426654 and rs16891982, previously reported to be fixed in Europeans (frequency < 0.90) and absent in East Asians and Africans [20], were used here to validate EUR. rs1800414 is limited to East Asians populations (frequency 0.50–0.60) and absent elsewhere, and was used to differentiate EAS from AFR [21].

Association between geographic groups and frequency of the variant allele for each polymorphism was analysed by least squares regression to generate *p* values and adjusted *r*^2^ values. Categorical comparisons of mean allele frequency between geographic groups were made using ANOVA (Tukey’s post hoc test). Analyses were weighted by cohort sizes. The *p* value threshold was adjusted for multiple testing using the Bonferroni method [41] to *p* < 0.001 for associations between variants and geographic region and *p* < 0.0001 for multiple comparisons between regions. Statistical analyses were performed using JMP (V13; SAS Institute Inc., Cary, NC, USA).

## Supplementary information

**Additional file 1:Supplementary material 1.** Further information on which cohorts gave data for each vitamin D-related variant and on UVB data collected for sample set locations.

## Data Availability

The datasets analysed during the current study are publicly available in the Alfred Frequency Database (ALFRED) and 1000 Genomes repositories, accessible through https://alfred.med.yale.edu/alfred/index.asp and www.ncbi.nlm.nih.gov/variation/tools/1000genomes/ respectively [34, 35]. The UVB data collected for sample set locations is also available via NASA’s web application, Giovanni (https://giovanni.gsfc.nasa.gov/giovanni/) [40].
